# Den Entry Behavior in Scandinavian Brown Bears: Implications for Preventing Human Injuries

**DOI:** 10.1002/jwmg.822

**Published:** 2015-02-13

**Authors:** Veronica Sahlén, Andrea Friebe, Solve Sæbø, Jon E Swenson, Ole-Gunnar Støen

**Affiliations:** Department of Ecology and Natural Resource Management, Norwegian University of Life SciencesPO Box 5003, Ås, NO-1432, Norway; Department of Biological Sciences, Goethe University Frankfurt, BioCampus–NiederurselMax-von-Laue-Straße 9, Frankfurt am Main, 60438, Germany; Department of Chemistry, Biotechnology and Food Science, Norwegian University of Life SciencesPO Box 5003, Ås, NO-1432, Norway; Norwegian Institute for Nature ResearchNO-7485 Trondheim, Norway; Department of Animal Ecology, Swedish University of Agricultural SciencesSE-901 83 Umeå, Sweden

**Keywords:** bear-human conflict, bear management, behavior, brown bear, den entry, pre-denning activity, Scandinavia, *Ursus arctos*

## Abstract

Encounters between Scandinavian brown bears (*Ursus arctos*) and humans that result in human injuries and fatalities typically coincide with den entry in October and November, and commonly occur near a den. Our aim was to determine when bears arrive at their dens, identify potential predictors of this event, document behavior and activity associated with this period, and attempt to explain the increased risk of bear-caused human injuries in this period. We analyzed global positioning system (GPS) location and activity data from brown bears in south-central Sweden, using generalized linear mixed models, statistical process control, and activity analyses. Bears arrived at their den sites between 6 October and 1 December. Timing varied by reproductive category, bear age, and year. Half of all bears significantly reduced their activity before arriving at the den area: on average 2,169 m away from the den and 1.8 days before arrival. The other half reduced their activity after arriving at the den area. The latter bears took longer time to reach hibernation activity levels, but we did not find a difference in the start date of hibernation between the 2 groups. Bears also appeared to be sensitive to disturbance in this period, with higher den abandonment rates than later in winter, particularly for males and for bears that had not visited their den sites previously. Den entry occurred from October to December, with high variability and poor predictability of its timing. Therefore, restricting hunting or other recreation activities to reduce risk of injury by bears and disturbing bears probably would be both impractical and ineffective. Our findings can be used to educate hunters about bear behavior at this time of year. Many people associate dens with an increased risk of a bear responding aggressively to disturbance to defend its den, but our results indicate that other behavioral, and possibly physiological, changes in this period also may be involved. © 2014 The Authors. The *Journal of Wildlife Management* published by The Wildlife Society.

Protection and careful management enabled the recovery of the Scandinavian brown bear (*Ursus arctos*) population from the few individuals that survived persecution at the end of the 1800s to a population that now exceeds 3,000 individuals (Swenson et al. 1995, Kindberg et al. 2011). The bear has enjoyed more support from the general public than other large carnivores in Sweden (i.e., gray wolves [*Canis lupus*] and wolverine [*Gulo gulo*]; Sandström and Ericsson 2009), possibly because of its status as a game species and low levels of livestock depredation. However, bears do injure and kill humans in Scandinavia (Swenson et al. 1999b*b*). Such incidents have increased with increasing number of bears, and perhaps more importantly, with increasing hunting quotas and harvest (Sahlén 2013), which has resulted in decreasing public support for the bear (Sandström and Ericsson 2009). If managers understand why some bear-human encounters lead to human injury, they can minimize the risk of human injury and fatalities and maintain public support for conserving the bear population.

A recent review of all known bear-caused human injuries and deaths in Scandinavia since 1977 documented that the risk of injury from an attacking bear is greatest from the end of September to mid-November, and that the vast majority of injured people were armed hunters (Sahlén 2013). During October–November, a large proportion of the incidents occurred at, or near, a winter den. This period of increased risk of injury coincides both with brown bear den entry (Friebe et al. 2001, Manchi and Swenson 2005) and the moose (*Alces alces*) hunting season, when large numbers of hunters are present in the forest. Experimental approaches in the study area by researchers simulating hikers have documented that most bears leave their daybed or foraging area when approached (Moen et al. 2012). However, the stalking, quiet behavior of hunters probably makes them more likely to surprise bears at close range than other groups of recreational forest users, exposing them to an increased risk of attack and injury. The greatest number of people use the forest in July and August, when berry-pickers, recreational users, and bird and bear hunters are present. Bear hunting has become increasingly popular since 2008, with a greater proportion of bears now being shot by specialized bear hunters before the start of the moose hunting season as opposed to opportunistically hunted by moose hunters (J. Kindberg, Swedish University of Agricultural Sciences, personal communication). Bear hunting attracts a large number of hunters, particularly during the early part of the bear hunting season, which begins on 21 August and lasts until 15 October, unless quotas fill earlier. Despite this, very few bear-inflicted injuries occur during the bear hunting season (Sahlén 2013). Part of the explanation might lie in changes in the bears' behavior near and at the den site during their behavioral and physiological preparation for winter.

The bear denning period in Scandinavia spans from October until May. Timing of den entry by bears is influenced by factors such as sex, reproductive status, and environmental conditions (i.e., first snowfall), as well as age and body size (Friebe et al. 2001, Manchi and Swenson 2005). A recent study of American black bears (*Ursus americanus*) in Alaska has shown that proximity to human activity and precipitation in early summer also may affect the timing of den entry (Baldwin and Bender 2010). Most previous research on brown bear denning chronology was based on very high frequency (VHF) radiotelemetry data, but the use of global positioning system (GPS) data provides detailed information both temporally and spatially. The high resolution of GPS data allows researchers to ascertain den locations, date of entry, duration of denning, and potential den abandonments with greater certainty than has been possible previously. These data are particularly useful for denning chronology when complemented with activity data from sensors measuring acceleration within the collars.

We used GPS and activity data based on accelerators from bears with confirmed den locations to describe changes in bear activity levels (movement independent of translocation) and their movement (translocation) to and around the den site, and to identify variables that may influence such movement. Our aim was to determine how brown bear movement and activity before and during the den entry period might explain the documented increase in the probability of aggressive behavior in encounters with humans during this period. In addition, we wanted to determine whether it was possible to predict when den entry, and thus these aggressive encounters, is most likely to occur.

## Study Area

We conducted this study in the Scandinavian brown bear population's southern reproduction area, which was located in south-central Sweden (61°N, 14°E). The area consists of gently rolling hills, and most of the area (>90%) lies below the timberline (approx. 750 m ASL; Dahle and Swenson 2003). The study area was within the northern boreal forest zone and dominated by Scots pine (*Pinus sylvestris*) and Norway spruce (*Picea abies*). The forest was a patchwork of tree monocultures and large clear-cuts due to intense forestry management. Information from 1999 shows that clear-cuts comprised approximately 8% of the forested areas and roughly 40% of the forest was <35 years old (Swenson et al. 1999a*a*). The area was sparsely populated by humans and limited to a few villages and single isolated cabins, many of which were only seasonally inhabited. Forestry management had generated an extensive road system of varying size and quality that ranged from unmaintained gravel roads to the paved national main road E45 (highway), which provided the main inland connection between north and south of Sweden (Nellemann et al. 2007).

The study area was situated on the border of 2 counties, where the bear population density was about 30 individuals per 1,000 km^2^ (Bellemain et al. 2005, Solberg et al. 2006). Harvest of brown bears was regulated by quotas set at the county level by the County Administration Boards. The area was popular for bear hunting, with a large number of guest hunters present during the first week of hunting, and quotas were typically filled within the first 2 weeks of the season. Moose hunting was also permitted in some parts of the area from the first Monday in September until the end of the month, with a 2-week break for the moose rut until the first Monday in October after which hunting was permitted in the entire area until the end of February. The highest hunting activity for moose was the period before the break.

## Methods

### Study Animals and Data Collection

Scandinavian Brown Bear Research Project personnel captured and handled bears during March–May using the methods for marking and capturing previously described (Arnemo et al. 2011, Fahlman et al. 2011). Project personnel captured offspring of radio-marked females, as well as previously unmarked adult and subadult bears. If the bear had not been followed from birth, age was determined by Matson Laboratory LLC (Miltown, Montana) by counting the annuli in a cross-section of a premolar root (Matson et al. 1993). The bears in this study were equipped with a GPS Plus-3 or GPS Pro-4 neck collar, which was fitted with a dual-axes motion sensor (activity sensor), VHF-transmitter, and a global system for mobile communications (GSM) modem (VECTRONIC Aerospace GmbH, Berlin, Germany). Bear capture was approved by the Swedish Environmental Protection Agency (permit Dnr 412–7327-09 Nv) and Uppsala's Ethical Committee on Animal Experiments (Djuretiska nämnden i Uppsala, approval number C47/9).

Collars were programmed to collect locations at 30-minute intervals during 1 August until 30 November (except for 2004 and 2005, when they had been programmed for 3-hr intervals), and once daily (at noon) from 1 December until 30 March. Activity sensors measured true acceleration in 2 orthogonal directions 6–8 times per second. The acceleration values were then averaged for each orthogonal direction separately during the time interval between 2 successive fixes over a recording interval of 5 minutes. We selected data from bears for which we had GPS location data and activity measurements before, during, and after hibernation (Aug until mid-Jun) in each year. We used the data from all bears (*n* = 45) to identify den sites and instances of den abandonment to ensure that we identified all dens used during the hibernation period. Potential den sites were identified using matched activity and GPS data (i.e., locations where the bear collar showed low activity (activity values that were lower than the activity threshold between active and passive behavior defined by Gervasi et al. 2006) and stationary behavior (clusters of consecutive GPS locations within a 50-m radius). Observers later confirmed the presence of a den with field visits and registered the habitat within a 50-m radius around the den opening and recorded evidence of predenning behaviors, such as digging attempts. For the analyses related to den entry, we selected the data from 1 August until at least 31 December for the bears whose dens we had visited.

We analyzed 90 den observations from 45 individuals (16 males [*n*_obs_ = 29], 29 females [*n*_obs_ = 61]) aged 2–18 years (median = 6 years) during 7 winters in 2004–2011. Den sites of females were further classified as those of females that were single, pregnant, or with young. Females with young were divided into females with cubs of the year (henceforth females with cubs), or females with yearlings (Table1). Based on the den entry date, as defined by the activity data (see below), we included only 1 first den per individual per season in the analysis, which resulted in 70 observations of den entry (Table1). We also excluded from the analysis 1 female whose collar recorded very low activity levels throughout the 2004–2005 season, and 1 male who denned outside the area in 2009–2010 because we did not have digitalized road maps.

**Table 1:** Data used in the study of denning behavior by Scandinavian brown bears in south-central Sweden from 2004–2010 related to individual bears (*n*) and observations of den entry (*n*_obs_), in total and for each bear category

Data selection category	All dens		First dens only	
	*n*	*n*_obs_	*n*	*n*_obs_
Individuals	45	90	45	70
Males	16	29	16	20
Females	29^a^	61	29	50
Single	10	12	10	11
Pregnant	21	39	21	31
With cubs	3	3	3	3
With yearlings	4	7	4	5
Age (years)				
Min.	2		2	
Max.	18		18	
Mean	7.6		7.6	
Median	6		6	
Dens	89^b^	90	70	70
Den attempts		4		
First dens		70		
Second dens		15		
Third dens		1		
Den abandonment	17	20	14	15
Attempted shifts		4	4	
Early shifts	11	13	11	12
Mid-season	3	3	3	3

a One female can be included in several categories as it is followed over several years.

b One bear used a previous first den as a second den 2 years later.

### Location Data

An initial visual assessment of the GPS location data in ArcGIS 10 (ESRI Inc. 2010) showed that bear positions around each den site during winter extended beyond 50 m only in cases of erroneous location points or cases of den abandonment; thus, we defined a 50-m radius of the den opening as the den site. This was also the area we assessed during field visits (see above). Because our primary purpose was to assess how bear behavior at the time of den entry could explain increased risk of human injury, we quantified how bears moved prior to entering the den. Experimental studies have revealed that bears in the study area tended to leave an area when observers were on average 115 m away if the bears were active (moving, foraging, etc.) and on average 67 m if they were passive (resting in a daybed; Moen et al. 2012). To assess the sensitivity of bears to humans during the den entry period, we defined the den area as the area within 150 m and we quantified how much time the bear spent within this area prior to hibernation. We distinguished between arriving at the den site or den area to begin hibernation and a visit to the den area. A visit occurred in the fall when a bear left the den site or area and stayed away for a minimum of 24 consecutive hours. We calculated the duration of each visit as the temporal difference between the first and last GPS location of that visit, and added the duration of all visits together for the total duration. Visits consisting of only 1 GPS-location were counted as 29 minutes in duration. Arrival at the den site was defined as the date of the first GPS location after which the bear did not leave the den area for more than 24 hours. Because GPS data can reveal if a bear was at a den site but not if it had entered the den, we used the term arrival. Arrival is a component of den entry, and we use den entry when discussing this period in more general terms. For each den site, we calculated distance to the nearest road of a particular class (roads were divided into classes according to size and quality) and settlement, which were divided into classes according to number and frequency of use (Table2) based on straight-line distances (m).

**Table 2:** Definitions of the variables used in the models to determine factors influencing denning behavior of Scandinavian brown bears in south-central Sweden from 2004–2010

Variable	Definition
Bear category	Bear reproductive class
Single female	Female unaccompanied by young during den entry, who did not give birth during the following winter
Pregnant female	Female unaccompanied by young during den entry, who gave birth during the following winter
Fwy	Female accompanied by dependent offspring (cubs of the year or yearlings)
FCOY	Female accompanied by cubs born during the previous winter (cubs of the year). Females with cubs in the text
FY	Female accompanied by cubs born 2 winters earlier (yearlings). Females with yearlings in the text
Male	Males
Den	A location where a bear has been stationary (i.e., not left for more than 24 consecutive hours at a time) for a minimum of 5 days
Attempt	A potential den location according to global positioning system (GPS) data, but field investigations did not reveal any den structure, only digging attempts or partial dens, or the bear reached hibernation activity at a subsequent den site
First den	The first den where the bear first reduced its activity below the threshold value
Second den	The new den after abandonment of a first den
Third den	The new den after abandonment of a second den
Den abandonment	The bear has left the den site and moved to a new location
Attempt shift	Abandonment of a den attempt
Early shift	Abandonment of a den before 14 December
Mid-season shift	Abandonment of a den between 15 December and 14 February
Late season shift	Abandonment of a den after 15 February (separated from den exit by the bear selecting a new confirmed den after abandonment)
Arrival at den area	The first GPS location within 150 m of the den where the bear does not leave for more than 24 consecutive hours prior to arrival within 50 m
Arrival at den site	The first GPS location within 50 m of the den, after which the bear is stationary for a minimum of 5 days, without being away from the site for more than 24 consecutive hours at a time
Hibernation date	The first day in a 7-day period where the activity does not go above the hibernation activity threshold (<22.8)
Visit	Time spent within 150 m of the den which is separated in time from arrival and other visits by at least 24 hr
First visit	First position within 150 m of the den site
Number of visits	Number of visits within 150 m of the den site
Total duration	Total time spent during visits within 150 m prior to arrival within 150 m
Number of days within 150 m	Total time spent within 150 m after arrival within 150 m until reaching the hibernation activity threshold
Roads	
E45	Paved main road through the area, the inland connection between south and north of Sweden (state road)
Main roads	Paved main roads within the district. Connects the largest communities (county roads)
Main gravel roads	High standard gravel roads. Connects larger roads and minor communities (county and communal roads)
Medium gravel roads	Gravel roads of good standard with a relatively constant but minor traffic. Typically connecting larger roads, minor communities and recreation sites, or used as a short-cut between larger roads (communal and private roads)
Minor gravel roads	Gravel roads of varying quality. The activities associated with these are occasional and unpredictable (e.g., forestry, recreation, berry picking, hunting and fishing; communal and private roads)
Railroad	Low activity railroad, mostly cargo, which runs largely parallel to the E45. Limited tourist traffic during summer
Settlements	
Type 1 & 2	Forest cabins; low and unpredictable activity; summer houses and hunting cabins; varying activity between and within seasons
Type 3	Permanent settlement throughout the year; single house to small communities (<50 inhabitants)
Type 4	Larger communities; villages and towns (>50 inhabitants)

All GPS location data are subject to 2 types of error: missing location fixes and location error (D'Eon et al. 2002), which are influenced by habitat, terrain, topography, fix intervals, and animal behavior (e.g., Cain et al. 2005, Heard et al. 2008 and references therein). Missing location fixes in our data set were recorded as 0 positions, and therefore easily identified and excluded, but location errors were more difficult to identify and eliminate, unless the location errors were very large (i.e., for our GPS data, we used a maximum speed threshold to filter out unlikely positions). The consequence of minor location error is that an animal may appear to be moving despite being stationary. In addition, bears tend to select denser habitats for their resting sites (Moe et al. 2007, Ordiz et al. 2011), which is a combination of cover and behavior that can decrease GPS fix rates (Heard et al. 2008). The number of 0 positions received during the den entry period indicates this is likely to be an even greater problem at den sites, because many den sites are underground or in very dense cover. The proportion of 0 positions can be an effective index for determining the timing of den entry from GPS data. However, because our interest was in the bear's behavior before it became inactive in its den, we used GPS data to define arrival at a den site and relied on activity data to determine when a bear became inactive, thus indicating a hibernating state.

### Activity Data

To categorize activity levels, we created individual, 5-min activity indices by summing the averaged acceleration values of the 2 orthogonal axes; indices ranged from 0–510. We defined a bear as active when its activity index was higher than 22.9. This threshold value was based on the first tests of the Vectronics dual-accelerated motion sensors reported by Gervasi et al. (2006). Hibernation is a physiological state, but we used prolonged inactivity in this period as a proxy for hibernation and use this term throughout this paper. We defined the start of hibernation (hibernation date) as the first day in autumn when the bear was active <1 hour per day (i.e., fewer than 12 5-min activity recordings >22.9 per day; Laske et al. 2011).

To quantify when activity changes were significant, we used statistical process control, which is commonly used for controlling industrial processes (Shewhart 1931). The basic steps of statistical process control are first to identify an “in control” or normal behavioral process, which here is a bear's activity pattern before denning. Based on normal behavioral data, we fitted a mean trend and estimated residual variance. Usually control borders are 2–3 standard deviations around the mean. The process is then allowed to run beyond the range of the normal behavioral data, and if the process (here the activity level) crosses the control borders, then the process is said to be “out of control.” For our purpose, a reduced activity level below the normal activity level indicated the start of predenning activity.

### Statistical Analyses

#### Predenning activity

Bears generally have a bimodal daily activity pattern with 2 activity peaks (early morning and late evening) and two activity lows (midday and middle of the night; Moe et al. 2007), with the amplitude and duration of the high and low periods changing with the time of year. Initial plotting of the 5-minute activity data revealed a reduction in activity and increase in duration of the daily low activity periods throughout the fall up to hibernation, but the bimodal activity pattern persisted. The bimodal daily activity pattern complicates determination of predenning activity. The mean trend in the bear activity data before denning could be estimated by a moving average estimate. However, low-activity periods within a high-activity period would make this impractical. Because we were most interested in the high-activity levels, we estimated a moving average of the upper 90th percentile of the activity data, rather than a moving average across all data. We set the width of the moving window to 150 consecutive data points. This resulted in a ragged curve, so we used a LOWESS smoother (Cleveland 1979) to produce a smoothed final activity curve.

**Figure 1:**
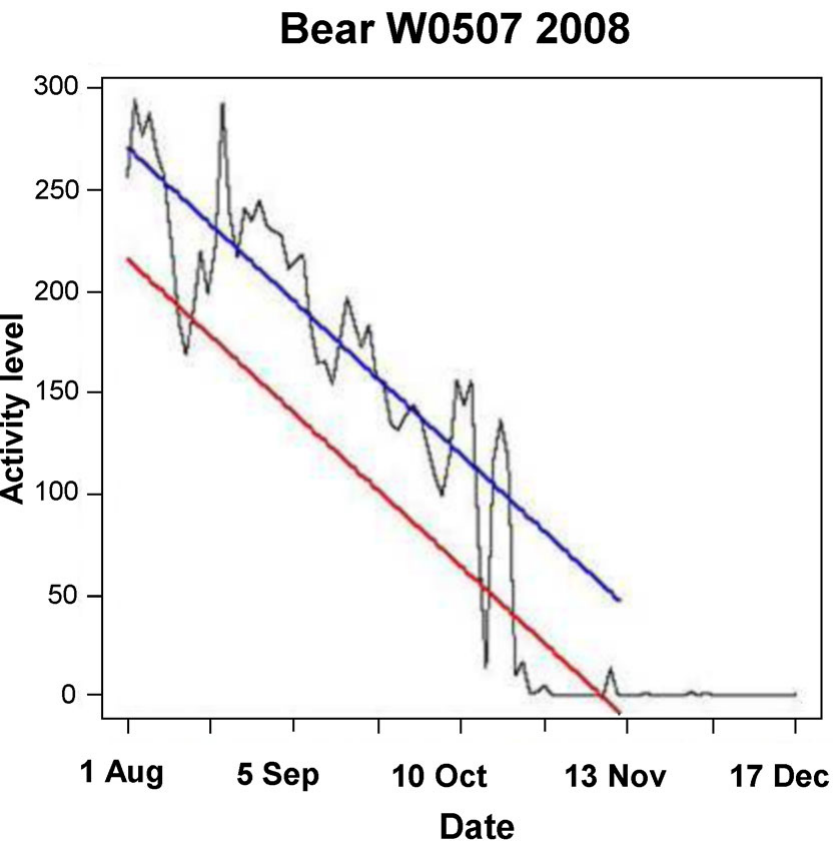
Example of pre-denning activity analysis on an individual Scandinavian brown bear in south-central Sweden in 2008. The blue line shows the bear-specific linear model of normal behavior data, and the red line is the lower control limit (LCL). We defined the start of predenning activity as the first time the activity value dropped below and remained under the LCL (20 Oct in this example). The observed 5-minute activity (black line) clearly shows the gradual reduction in activity, the variation in activity, and hibernation activity levels.

We used activity curves from 67 bears as input into the statistical process control. As a first step, we defined a period of in control (normal behavior) observations for each individual. This period started long before denning was expected to start. Then we used the data from the period of normal behavior data for all 67 bears as observed responses in a linear mixed model. Let *Y_ij_* denote activity value of the *j*th observation during the period of the in control activity data (smoothed curve) for bear number *i*. Further, let *X_ij_* be the time corresponding to the observed response, and *a_i_* be the random intercept term associated with bear number *i* (for *i *= 1, …, 24). We assumed the random variables to be normally distributed with zero mean and variance 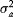 hence, we assumed 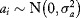. Further, we defined another random term as the random effect of time for each bear, with the assumption 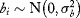. The model we assumed for the data was 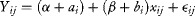 where *α* and *β* are the intercept and slope parameters common for all bears and 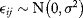 is the noise term. We estimated the fixed and random parameters using the nlme- package in R, based on the restricted maximum likelihood (REML) method for estimation. This approach fits a bear-specific linear model to the normal activity data, but we estimated the noise variance *σ*^2^, which is of particular interest, based on all 67 bears. Upon estimation of the linear model and the noise variance, we defined a lower control limit for bear *j* as:


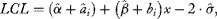


which represented the fitted linear model for the individual bear minus 2 estimated standard deviations. We defined the point in time of the start of predenning activity as the first time the activity value dropped below the *LCL* in the expected denning entry period (Fig. 1).

We were unable to identify behavioral changes in 2 bears because of low activity levels and an associated gradual change, such that the method could not identify a definite point in time where activity values indicated a change to predenning activity. Consequently, the predenning activity dates were uncertain for both bears. For a third individual, the predenning activity date occurred after the hibernation date. All 3 bears had potential denning attempts before the hibernation date, but either we could not confirm dens during field visits, or dens were confirmed, but the bear switched dens before the hibernation date (thus, the location did not qualify as a den). Therefore, we excluded these 3 observations from further analysis.

Bears either began predenning activity before arriving at the den area (PDAB) or after arriving at the den area (PDAA). We therefore tested for differences in mean arrival dates in the den area, predenning activity date, hibernation date, and time between predenning activity and hibernation dates between PDAA and PDAB bears using Welch's tests (accounting for uneven sample sizes and variances) on transformed variables (log or square root to normalize residuals), or Wilcoxon rank-sum tests (for variables whose residuals could not be effectively normalized). We tested if males or females were more likely to begin their predenning activity before or after arrival at the den site using a Chi-square test of association with Yates' correction for continuity.

#### Movement at and near den sites

Because of the high number of potential explanatory variables in relation to the relatively low number of den entry observations, our models risked non convergence and false convergence (over specification of the model). Therefore, we first ran a principal component analysis (PCA), using the statistical programming language and environment R version 2.14.1, and the PCA package (FactoMineR library, R Development Core Team 2011), where we included all continuous variables to evaluate whether any variables were clustered. Because the PCA is sensitive to non-normality, we log-transformed all continuous variables with a non-normal distribution to normalize the data (Table S1, available online at http://www.onlinelibrary.wiley.com). We excluded variables that could not be normalized from the PCA. We selected variables from the resulting dimensions and included those seen as relevant in the subsequent generalized linear mixed models (GLMMs) using the lmer/glmer package (lme4 library, R Development Core Team 2011). We included the individual as a random variable in all GLMM models to account for repeated sampling of individuals. We used the model.dredge package (MuMIn library, R Development Core Team 2011) to identify the best candidate models. Model dredging has been criticized as a “fishing expedition,” which can produce spurious results (Burnham and Anderson 2002); however, such an approach can be useful for observational studies with a high number of potential explanatory variables (e.g., Hegyi and Garamszegi 2011, Symonds and Moussali 2011). When using an information-theoretic approach, it is important to select explanatory variables with care, as the results must always be considered in relative terms (i.e., selection of variables with little biological relevance could still generate a best model). For our models, we selected potential explanatory variables based on what had been previously substantiated in other studies on den entry and den selection (e.g., Friebe et al. 2001, Manchi and Swenson 2005, Elfström et al. 2008, Elfström and Swenson 2009, Baldwin and Bender 2010). Because all variables have a biological rationale, support from previous research, or both, we are confident of their biological and ecological relevance, which further reduced the risk of finding nonsensical candidate models.

Model selection is often based on the calculation of Akaike's Information Criterion (AIC) value, where the model with the lowest AIC is typically considered the best model and the difference between the AIC value of the top model and other candidate models is known as the ΔAIC (Burnham and Anderson 2002). Candidate models with ΔAIC <2 are generally considered as equally good, whereas models with a ΔAIC <6 should not be discounted (Burnham and Anderson 2002, Richards 2005). In the case of small sample sizes, this is accounted for by calculating corrected AIC (AIC*_c_*) values, which was the case in our analyses (Sugiura 1978 in Garamszegi 2011). Therefore, we selected candidate models with a ΔAIC*_c_* <6 from the model dredging results, and used them to calculate model and variable weights. Akaike weights can be interpreted as the probability of a given model to be the best approximating model (Symonds and Moussali 2011), and thus we calculated AIC*_c_* weights for each candidate model. To assess the relative importance of each explanatory variable on each response variable, we summed the weights of the models in which a given variable was included, to obtain variable weights (Symonds and Moussali 2011).

To determine which variables influenced timing of arrival at a den site, we ran a GLMM, with the full model including the following variables (transformed where applicable): reproductive category  + year + age + previous visits to den area (Y/N) + distance to main roads + distance to main gravel roads + distance to minor gravel roads + distance to settlements type 1 and 2 + distance to settlements type 3 + distance to the E 45 highway.

To determine which variables influenced the number of days bears spent within 150 m of the den (i.e., the den area) prior to reducing activity below the hibernation threshold, we ran a GLMM with the original model including the following variables (transformed where applicable): reproductive category + year + age + arrival date at the den site + distance to main roads + distance to main gravel roads + distance to minor gravel roads + distance to settlements type 1 and 2 + distance to settlements type 3 + distance to the E 45 highway.

To determine factors that may have influenced den abandonment, we ran a GLMM with a binomial link function. We included the following variables: sex + age + year + arrival date at den site + distance to settlements type 1 and 2 + distance to settlements type 3 + distance to main roads + distance to main gravel roads + distance to E 45 highway + time spent in the den area + time spent within the den area + previous visits to den area (Y/N). We also included the interactions sex:age and sex:previous visits. The model selection for den abandonment produced many models with ΔAIC*_c_* between 2 and 6 that varied very little in weight from each other, and did not contain any additional variables. We therefore elected to calculate weights based on the candidate models with ΔAIC*_c_* <2.

## Results

### Predenning Activity

Bears began predenning activity on 22 October ± 11 days (mean ± SD, median: 23 Oct). Bears began predenning activity before arriving at the den area (PDAB) on 35 of 60 occasions (58%), and after arriving at the den area (PDAA) on 25 occasions (42%). The PDAB bears began predenning activity on 20 October ± 10.6 days (median: 20 Oct) and the PDAA bears began predenning activity on 25 October ± 10.3 days (median: 23 Oct).

The PDAB bears began predenning activity on average 2,164 ± 1,690 m from the den (median: 1,662 m, range: 30–7,310 m). One observation of 1 bear at a distance of 30 m was included, because our arrival definition did not include temporary stays in the den area that were separated by more than 24 consecutive hours. For all other PDAB bears, predenning activity began 1.8 ± 1.8 days (median: 1.3, range: 0.1–7.9 days) before arriving at the den area and 5.7 ± 3.5 days (median: 5.2 days, range: 1.3–16.5 days) before hibernation. These bears spent 4.0 ± 3.4 days (median: 3.0 days, range: 0–14.9 days) in the den area before hibernation, of which 3.6 ± 3 days (median: 3.0, range: 0–12.4 days) were spent at the den site. Most PDAB bears (66%) had visited the den area prior to the final arrival (*n* = 23, average visits: 2.3 ± 2.3 visits, median: 1, range: 1–10).

The PDAA bears began predenning activity on average 175 m ± 430 m from the den (median: 16 m, range: 2–1,741 m). One observation at 1,741 m was included, because our arrival definition allowed for stays outside the buffer area that lasted less than 24 consecutive hours. Predenning activity began 1.5 ± 1.4 days (median: 0.95, range: 0.09–5.4 days) after arriving at the den area, and 3.8 ± 3.7 days (median: 3.1, range: 0.01–14.1 days) before hibernation. These bears spent 5.4 ± 4 days (median: 4.8, range: 0.9–16.7 days) in the den area before hibernation, whereof 4.5 ± 3.1 days (median: 4.1, range: 0.9–12.9 days) were spent at the den site, and 72% had visited the den area prior to final arrival (*n* = 7, average visits: 2.9 ± 2.1, median: 2, range: 1–7).

The PDAB bears began predenning activity farther from the den than PDAA bears (Wilcoxon rank-sum test: W = 51, *P *< 0.001). We did not find a difference in timing of hibernation between the 2 categories of bears (Welch's *t*-test: *t*_52.076_ = 0.9797, *P *= 0.30); however, PDAB bears tended to begin predenning activity earlier than PDAA bears (Welch's *t*-test: *t*_52.665_ = 1.7351, *P *= 0.09, mean PDAB = 20 Oct, mean PDAA = 25 Oct). Time spent at the den area before hibernation also did not differ between types of bears (PDAB: 4.0 ± 3.4 days, PDAA: 5.4 ± 4 days, Wilcoxon rank-sum test: W = 338, *P *= 0.138); however, PDAB bears had shorter time between predenning activity and hibernation than PDAA bears (Wilcoxon rank-sum test: W = 250, *P *< 0.005). We did not find significant differences between PDAB and PDAA bears in number of visits to the den site (Wilcoxon rank-sum test with continuity correction: W = 538, *P* = 0.13). Females were more likely to be PDAB bears than males (28/46 females, 7/14 males, Chi square test of association with Yates' correction for continuity: 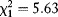, *P* < 0.025).

### Timing of Arrival and Movement at and near Den Sites

Bears arrived within 150 m of the den (den area) on average on 23 October ± 11.1 days (range: 6 Oct–30 Nov), spending 4.6 ± 3.8 days (max = 16.8 days) in the den area before hibernation. In 22 of 68 den entry events, bears did not visit the den area before arriving at the den area to stay. The others visited the den area 2 ± 2.2 times (range: 1–10 times), spending in total 13.8 ± 22.6 hours in the area during visits (range: 29 min–4.5 days) prior to their final arrival.

Bears arrived within 50 m of the den (den sites) on 24 October ± 11.4 days (mean ± SD, range: 6 Oct–1 Dec). They spent 4.0 ± 3.2 days at the den site (max = 14 days) before hibernation on 28 October ± 12.5 days (range: 6 Oct–15 Dec). On 3 occasions, bears arrived at their den sites with activity levels already below the hibernation threshold (i.e., arrival at the den site and entry was set to the same time). These bears had either previous den attempts or smaller clusters of GPS locations that did not fit the den criteria before arriving at the den area and den site. Their activity values before and during hibernation corresponded to that of other bears, thus indicating that the early reduction in activity levels was not an artifact of the activity sensors. As previously mentioned, these 3 individuals had reduced their activity before arriving at the den sites, indicating that the smaller clusters of GPS locations may have been early den attempts, despite our inability to find dens or partial dens at the locations.

### Factors Affecting Timing of Arrival at the Den Site

**Figure 2:**
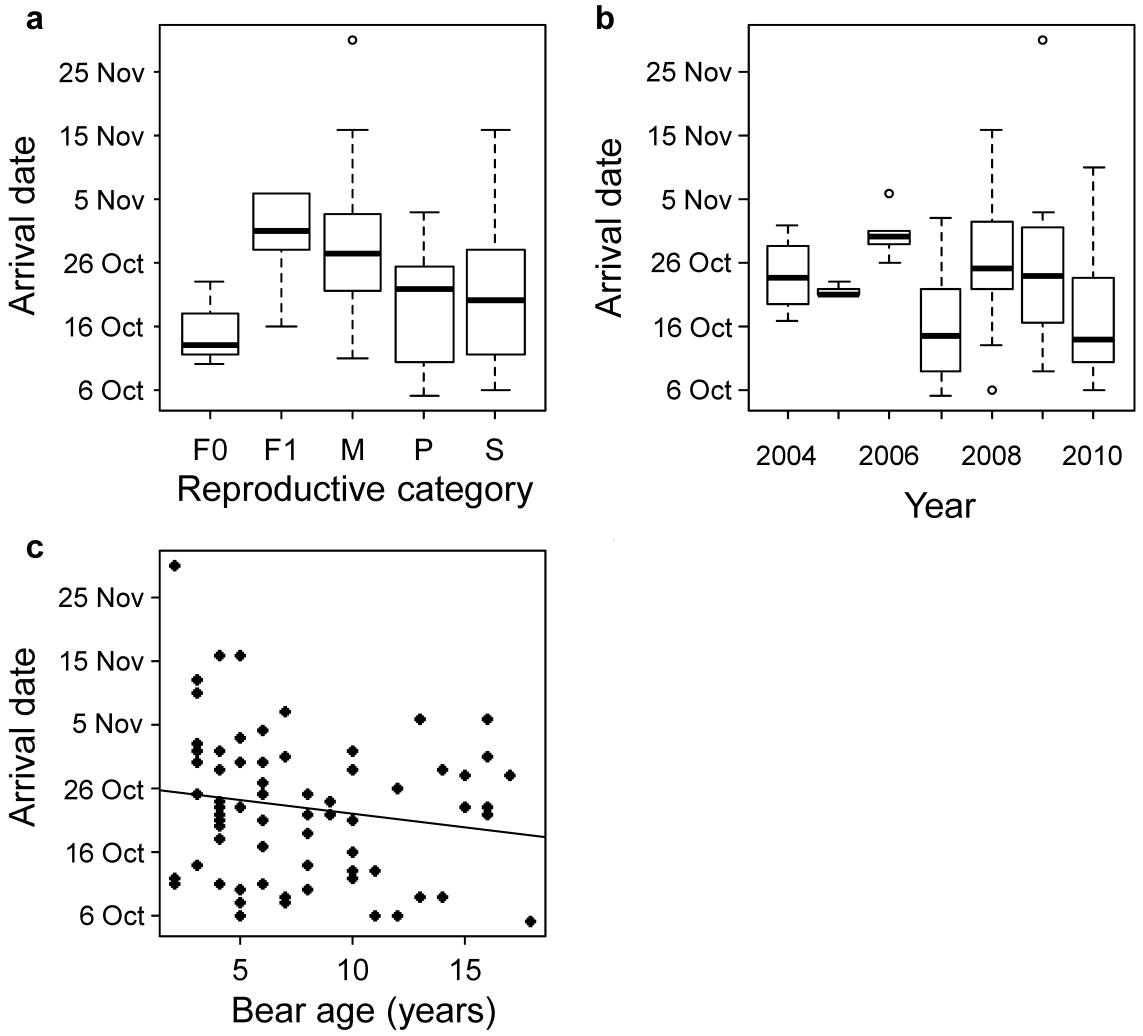
Arrival at the den site in Scandinavian brown bears in south-central Sweden by (a) reproductive category (F0 = females with cubs [*n* = 3], F1 = females with yearlings [*n* = 5], M = males [*n* = 19], P = pregnant females [*n* = 30], and S = single females [*n *= 11]), (b) year during 2004–2010, and (c) age (years).

Model dredging generated 5 candidate models with ΔAIC*_c_* <2 and 29 candidate models with ΔAIC*_c_* <6 (Table3). Variable weights indicated year and reproductive category as the main factors deciding the timing of arrival at a den site (Table3). Timing of arrival at the den site was earlier in 2007 and 2010 than the other years (Fig. 2b). Pregnant females (*n* = 30, median date of arrival: 22 Oct), single females (*n* = 11, median date of arrival: 20 Oct), and females accompanied by cubs of the year (*n *= 3, median date of arrival: 13 Oct) arrived at their dens earlier than males (*n* = 19, median date of arrival: 26 Oct) and females with yearlings (*n *= 5, median date of arrival: 31 Oct; Fig. 2a).

**Table 3:** Factors affecting timing of arrival of Scandinavian brown bears at the den site in south-central Sweden from 2004–2010. Blank cells show that the variable is not included in the candidate model, +indicates that a categorical variable is included in the candidate model, and numbers show the relationship between the intercept and the numerical variable in the candidate model

Model intercept	Variables									df	logLik^d^	AIC*_c_*^e^	ΔAIC*_c_*	Model weight
	Reproductive category	Age	E45^a^	Minor dirt roads	Settlement 1 & 2^b^	Settlement 3^c^	Main dirt roads	Year	Previous visits					
297.2	+	−3.30		5.82	5.86	−6.86		+	+	18	−192.85	435.70	0.00	0.17
295.2	+	−3.24		6.10	6.11	−7.16		+		17	−194.79	435.80	0.16	0.16
293.1	+			6.04	6.26	−8.06		+	+	17	−195.64	437.50	1.85	0.07
298.3	+	−3.29	−0.10	5.76	5.81	−6.78		+	+	19	−191.84	437.50	1.86	0.07
295.6	+	−3.24	−0.03	6.08	6.09	−7.14		+		18	−193.80	437.60	1.89	0.07
291.4	+			6.27	6.47	−8.29		+		16	−197.55	437.80	2.09	0.06
308.3	+	−3.57		5.18		−3.54		+	+	17	−196.23	438.70	3.03	0.04
305.6	+	−4.49		6.74	3.41	−7.43	0.12	+	+	19	−192.57	439.00	3.30	0.03
295	+		−0.19	5.92	6.18	−7.91		+	+	18	−194.61	439.20	3.52	0.03
306.3	+	−3.51		5.51		−3.75		+		16	−198.28	439.20	3.57	0.03
289.4	+	−4.04		4.89	2.51			+	+	17	−196.52	439.30	3.62	0.03
292.6	+		−0.12	6.20	6.42	−8.21		+		17	−196.54	439.30	3.66	0.03
301.5	+	−4.28		7.15	4.08	−7.93	0.11	+		18	−194.81	439.60	3.91	0.02
286.1	+	−4.00		5.19	2.67			+		16	−198.60	439.90	4.21	0.02
310.6	+	−3.53	−0.25	5.01		−3.37		+	+	18	−195.19	440.30	4.67	0.02
312.6	+	−3.62			4.33	−5.01		+	+	17	−197.07	440.40	4.71	0.02
312.1	+	−4.80		6.52		−5.60	0.13	+	+	18	−195.21	440.40	4.72	0.02
298.4	+	−4.00		4.77				+	+	16	−198.89	440.50	4.79	0.02
312.9	+	−4.48	−0.70	6.30	3.09	−6.96	0.12	+	+	20	−191.37	440.60	4.95	0.01
307.9	+	−3.48	−0.18	5.40		−3.65		+		17	−197.26	440.80	5.10	0.01
294.5	+	−3.95	−0.47	4.62	2.50			+	+	18	−195.44	440.80	5.17	0.01
304.6	+			5.35		−4.59		+	+	16	−199.18	441.00	5.36	0.01
306.9	+	−4.26	−0.54	6.85	3.85	−7.60	0.11	+		19	−193.70	441.20	5.57	0.01
295.4	+	−3.95		5.10				+		15	−201.01	441.20	5.58	0.01
310.2	+	−3.53			4.66	−5.38		+		16	−199.32	441.30	5.64	0.01
290.4	+	−3.92	−0.41	4.99	2.65			+		17	−197.55	441.30	5.68	0.01
308.7	+	−4.62		6.97		−5.78	0.12	+		17	−197.61	441.50	5.80	0.01
302.8	+			5.64		−4.76		+		15	−201.19	441.60	5.95	0.01
318.5	+	−3.50	−0.73		4.22	−4.61		+	+	18	−195.84	441.60	5.98	0.01
Variable weight	1.00	0.80	0.28	0.96	0.83	0.91	0.11	1.00	0.54					

a Distance to paved main road through the area, the inland connection between south and north of Sweden (state road).

b Distance to forest cabins; low and unpredictable activity; summer houses and hunting cabins; varying activity between and within seasons.

c Distance to permanent settlement throughout the year; single house to small communities (<50 inhabitants).

d Log likelihood.

e Corrected Akaike's Information Criterion.

Bears arrived earlier at den sites that were closer to minor gravel roads. In contrast, bears tended to arrive later at den sites that were closer to small permanent settlements. However, both these effects were weaker. Older bears tended to arrive at their den sites earlier than younger bears (Fig. 2c). The differences between bear arrival date for the visited and not visited den sites were small and variable weights were minimal.

### Time Spent in Den Area Before Hibernation

**Figure 3:**
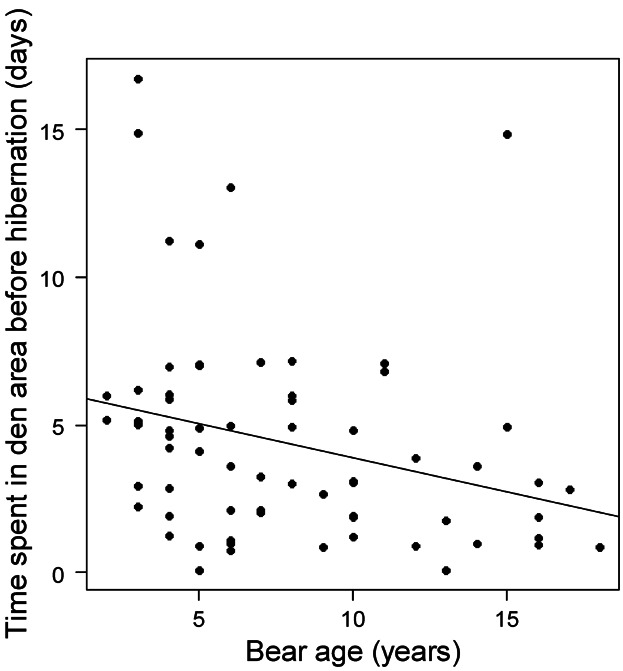
Number of days spent within 150 m of the den (den area) in relation to age (years) of Scandinavian brown bears in south-central Sweden during 2004–2010.

The top model for the number of days spent in the den area prior to hibernation, and the only model with ΔAIC*_c_* <2, included only age as a variable (Table4). Younger bears spent more time in the den area than older bears (Fig. 3). Age was the most influential variable according to the weights; all other variables had very little effect on time spent in the den area prior to hibernation.

**Table 4:** Factors affecting number of days Scandinavian brown bears spent in the den area (within 150 m of den) before hibernation activity in south-central Sweden from 2004–2010. Blank cells show that the variable is not included in the candidate model, +indicates that a categorical variable is included in the candidate model, and numbers show the relationship between the intercept and the numerical variable in the candidate model

Model intercept	Variable							df	logLik^d^	AIC*_c_*^e^	ΔAIC*_c_*	Model weight
	Age	E45^a^	Minor gravel roads	Settlement 1 & 2^b^	Settlement 3^c^	Main gravel roads	Visits					
0.98	−0.26							4	−38.39	85.4	0	0.43
0.14	−0.30	0.10						5	−38.37	87.7	2.30	0.14
0.42	−0.27			0.17				5	−38.77	88.5	3.09	0.09
0.49								3	−41.10	88.6	3.15	0.09
1.03	−0.24						+	5	−39.15	89.3	3.85	0.06
0.90	−0.26				0.02			5	−39.27	89.5	4.09	0.06
1.10	−0.26		−0.04					5	−39.36	89.7	4.27	0.05
0.65	−0.27					0.01		5	−39.78	90.5	5.11	0.03
0.61							+	4	−41.31	91.3	5.83	0.02
−0.23	−0.31	0.10		0.12				6	−39.00	91.4	5.95	0.02
Variable weight	0.88	0.16	0.051	0.11	0.06	0.03	0.10					

a Distance to paved main road through the area, the inland connection between south and north of Sweden (state road).

b Distance to forest cabins; low and unpredictable activity; summer houses and hunting cabins; varying activity between and within seasons.

c Distance to permanent settlement throughout the year; single house to small communities (<50 inhabitants).

d Log likelihood.

e Corrected Akaike's Information Criterion.

### Den Abandonment

**Figure 4:**
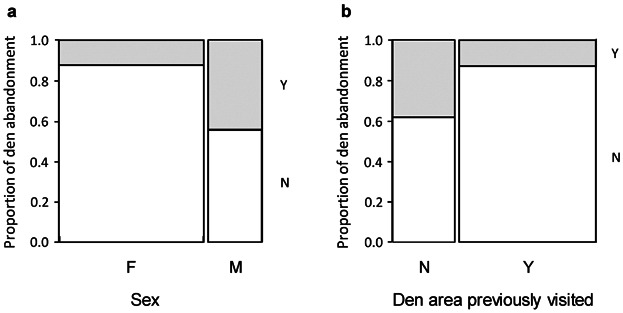
Proportion of den abandonment in (a) male and female Scandinavian brown bears, and (b) among Scandinavian brown bears in relation to whether they previously visited the den area (yes [Y] or no [N]). We recorded den abandonment in a population in south-central Sweden from 2004–2010.

Bears abandoned their first dens in 15 of 68 (22%) denning events. Most den abandonments occurred early in the denning period, with only 3 abandonments occurring after 15 December (Table1). Sex and prior visits to the den area by the bear were the most influential variables (Table5). Males abandoned their dens more frequently than females (Fig. 4a), and bears that made prior visits to the den area abandoned their dens less frequently than bears that did not (Fig. 4b). All other variables had low variable weights, indicating very little effect on whether or not bears abandoned their dens.

**Table 5:** Factors affecting den abandonment of Scandinavian brown bears in south-central Sweden from 2004–2010. Blank cells show that the variable is not included in the candidate model, +indicates that a categorical variable is included in the candidate model, and numbers show the relationship between the intercept and the numerical variable in the candidate model

Model intercept	Variable								df	logLik^c^	AIC*_c_*^d^	ΔAIC*_c_*	Model weight
	Arrival at den site	Minor gravel roads	Age	E45^a^	Settlement 3^b^	Time in den area	Sex	Visited					
−1.16							+	+	4	−29.63	67.9	0	0.17
−0.55						−1.16	+	+	5	−28.62	68.2	0.30	0.14
0.50		−8.00E−05				−1.47	+	+	6	−27.69	68.8	0.86	0.11
−1.97							+		3	−31.36	69.1	1.19	0.09
−0.58		−5.18E−05					+	+	5	−29.16	69.3	1.39	0.08
1.61				−0.32			+	+	5	−29.16	69.3	1.39	0.08
−1.85			0.39				+	+	5	−29.40	69.8	1.87	0.07
2.07				−0.30		−1.11	+	+	6	−28.21	69.8	1.89	0.07
3.40					−1.08	−1.30	+	+	6	−28.21	69.8	1.90	0.07
1.43					−0.72		+	+	5	−29.42	69.8	1.90	0.06
−8.36	0.03					−1.32	+	+	6	−28.23	69.8	1.93	0.06
Variable weight	0.06	0.19	0.07	0.15	0.13	0.45	1	0.91					

a Distance to paved main road through the area, the inland connection between south and north of Sweden (state road).

b Distance to permanent settlement throughout the year; single house to small communities (<50 inhabitants).

c Log likelihood.

d Corrected Akaike's Information Criterion.

## Discussion

Bears gradually reduced their activity during autumn, but we were able to statistically identify marked activity reductions—predenning activity—before they reached an inactive state and began hibernation. Approximately half of the bears reduced their activity while still far away from their dens, whereas the other half arrived at their den areas before reaching predenning activity. Females tended to be more likely to reach predenning activity before arriving in their den area, but the timing of predenning activity possibly depends on other factors, such as body size, individual condition, or possibly reproductive status (pregnant, accompanied by cubs, or single). Those that had already begun predenning activity before arriving at the den area reached hibernation faster than those that began predenning activity after arriving, but we did not find a difference in the actual timing of hibernation.

The pattern and timing of arrival at the den was similar to what has been previously reported for our study population, including the effects of reproductive category, age, and year (Friebe et al. 2001, Manchi and Swenson 2005). Single females, pregnant females, and females with cubs arrived at their den sites earlier than males and females with yearlings. We did not group females with cubs and females with yearlings as females with young in our analyses because they had large differences in the timing of arrival at their den sites, with females with yearlings more resembling males in their timing (Fig. 2). Friebe et al. (2001) did not detect an effect of age on entry dates for female brown bears (although denning duration increased with female age), but we detected a tendency for younger bears to arrive at den sites later than older bears. However, we did not analyze for differences within and between sexes in this respect, and age possibly affects male and female timing of arrival differently, as it does for duration of denning (Manchi and Swenson 2005). We also found yearly variations in timing of arrival at the den, which agrees with previous findings (Manchi and Swenson 2005). We did not include any weather or environmental variables in our analyses, because of the already high variable-to-observation ratio, but other studies have documented effects of environmental variables (e.g., food availability; Van Daele et al. 1990, Schooley et al. 1994), snowfall, and snow cover (Craighead and Craighead 1972, Reynolds et al. 1976, Servheen and Klaver 1983, Manchi and Swenson 2005) on den entry. Although this may be less important for pregnant females (Friebe et al. 2001), interannual variations in the onset of winter are the most likely explanation for the differences we documented.

Baldwin and Bender (2010) documented that bears entered their dens earlier when closer to roads and hypothesized that this may be due to increased access to food sources near roads, which allowed them to gain enough fat reserves to den early. We also found some lesser effects of distance to human activity, suggesting bears arrived at their den sites earlier when closer to minor gravel roads and smaller permanent settlements. Elfström and Swenson (2009) documented a tendency for adult males to den farther from plowed roads and permanent settlements. In our study, males arrived at their den sites later than other bears. Although we did not analyze for interactions between human infrastructure and reproductive category, the effects of distance to roads and settlements may actually be an effect of social organization in den selection (i.e., avoidance of dominant males; Elfström and Swenson 2009). Dumpsites for slaughter remains tend to be associated with minor gravel roads, however, and are typically used by local hunting teams on a yearly basis to dispose of hides and bones from the moose hunt (Sahlén 2006). Future research could document the presence and size of such sites in our study area to examine any potential effects they may have.

How long a bear spent in the den area before hibernation was related mainly to the age of the bear, with older bears spending less time in the den area than younger bears. This may be an effect of older bears' greater experience and familiarity with their home range. Manchi and Swenson (2005) documented that distance between an individual's dens in successive years was short for adult males and females irrespective of age, indicating that the same general area tended to be used for denning year after year, but that young male bears had long distances between successive years' dens, because of the subadult males' dispersal behavior.

The den abandonment rate we documented (22%) was higher than the 9%, based on VHF radio telemetry, reported previously for our study area (Swenson et al. 1997). However, the greater location accuracy and sampling frequency of the GPS data allowed us to record movements on a finer scale than when using VHF data. Most den abandonments in this study occurred early in the denning season; only 4% occurred after mid-December. This pattern, although different in effect size, is also in agreement with Swenson et al. (1997). These finer location shifts early in the denning season were probably less evident when relying on VHF tracking alone. Thus, we do not conclude that den abandonment rates have increased.

Males, and bears that had not visited their den area before final arrival, regardless of sex, were more likely to abandon their dens and most abandonments occurred early in the denning period. Given that the majority of den abandonments appear to be the result of human disturbance (Swenson et al. 1997, Linnell et al. 2000), this is also likely to be the cause for the den abandonments we documented in this study. The moose-hunting season starts at the end of September and is most intense during October and beginning of November, and forestry activities occur year round in the area. Both activities have great potential for disturbing bears, especially moose hunting, which often involves unleashed baying dogs. Bears that had visited the den area previously may be aware of most of the regular disturbances that occur and are therefore either used to them, or have already selected against such disturbances when choosing their den site. We know from small- and large-scale studies that adult males avoid human activity to a greater extent than other categories of bear (Nellemann et al. 2007, Elfström et al. 2008). Additionally, males are more likely to den in open nest dens, which could also make them more vulnerable to disturbance (Elfström and Swenson 2009). However, males' greater likelihood of abandonment may also be an effect of their better ability to bear the cost of abandonment (Beale and Monaghan 2004), due to their greater body size and fat reserves. Abandonment is particularly costly for pregnant females, which are more likely to lose their cubs than pregnant females that did not abandon their dens (Swenson et al. 1997). The cost of abandonment is likely to increase later in the winter season, when the bears are deeper in hibernation and the snow cover makes locating new suitable dens difficult (Evans et al. 2012). This, and less human activity in the forests, may explain the lower den abandonment rates documented in late winter.

When interpreting our den abandonment results, one must consider that responding to human disturbance by leaving may be more prevalent among captured and handled bears than among non-captured bears because of prior negative experience with humans. Changed behavioral responses to humans is an inherent problem in any wildlife research project involving captured and handled animals. The results from our experimental approach studies (Moen et al. 2012, Ordiz et al. 2013, Sahlén 2013) compared to anecdotal descriptions of encounters with non-captured bears by field staff and local residents suggest that they respond to human encounters similarly to radio-marked bears. Therefore, we conclude that human disturbance near den sites probably affected captured and non-captured bears similarly.

### Predenning Behavior and Human–Bear Interactions

One of our important findings is that bears do not have to be at their den site to begin predenning activity; in fact, half of the bears in our study were often kilometers away from their final den location when their activity levels dropped significantly. This means that many bears were moving in this lowered activity state for almost 2 days before arriving at their den area. Whether this activity state is only behavioral or also physiological cannot be assessed in this study, but it raises the question of whether bears in this activity state away from their den may respond similarly to meeting a human as would a bear in this state near its den.

Studies of rodent species have shown that animals faced with threats respond in a continuum of defensive behaviors, ranging from escape to fight and attack (Blanchard and Blanchard 1989). The fight response is typically triggered when animals have a limited ability to flee and the threat is close to the animal (Eilam 2005). Bears undergo a series of physiological changes during the hibernation period, including a decrease in body temperature (Nelson et al. 1983). Although the onset of hibernation is associated with environmental cues (Craighead and Craighead 1972, Reynolds et al. 1976, Schooley et al. 1994, Friebe et al. 2001, Manchi and Swenson 2005), brown bears and other mammals tend to begin hibernation even in absence of such cues, indicating a molecular genetic mechanism (Carey et al. 2003). The reduction in activity we documented in the bears did not depend on having settled into a den, which suggests that physiological changes affecting the bears' behavior may begin before this time. Studies on ectotherms, such as lizards and snakes, have shown a relationship between decreasing temperatures and increasing use of fight, rather than flight, as defensive behavior (e.g., Hertz et al. 1982, Crowley and Pietruszka 1983, but see Keogh and DeSerto 1994). This is because low body temperature impairs the ability to move, particularly on aspects of speed and endurance (Bennett 1990) but less so on the ability to defend themselves aggressively (Herrel et al. 2007). Body size may further affect whether or not an animal chooses to defend itself because an aggressive response may be ineffective as a defensive strategy for smaller individuals, whose best defense is then still to flee, even at diminished capacity (Cury de Barros et al. 2010). Muscle function in both endo- and ectotherms are affected by temperature, with lowered function associated with low body temperature (Bennett 1984). This could mean that brown bears react more aggressively to disturbance not because they are defending themselves at or near a den, but because their physiological state prevents them from using escape as an effective defensive mechanism. Injury rates on humans caused by bears increase during the den entry and moose hunting period (Sahlén 2013). Although a large part of the explanation for this lies in a high presence of moose hunters in the woods, who are behaving in a way that predisposes them to surprise encounters with wild animals, including bears, the physiological state of the bear during human encounters in this period may contribute to the outcome (Sahlén 2013). The use of hunting dogs may further affect the bears' behavior, either by cornering them in a den or by persistently following them in a prehibernation state. Whether or not this is the case requires further studies.

## Management Implications

Most bear-caused human injuries in Scandinavia occur during the moose-hunting season, which is concurrent with the bears' den entry period, and hunting activity, use of dogs, and presence of a den are the most common factors associated with bear-caused injuries in October and November. A high predictability in the timing of den entry could have permitted managers to use hunting restrictions during a limited period to reduce the risk of injury to humans and the risk of disturbance to the bears. However, we documented a very long time span over which den entry occurs (range: 6 Oct–15 Dec), and a high variability in its timing. This makes it difficult for managers to impose any such restrictions on recreation or hunting. Hunting restrictions lasting for the duration of the den entry period would certainly not be supported by hunters and would therefore be unlikely to be effective in reducing either risk of injury or disturbance. Reducing the moose hunting period could also interfere with moose management objectives. We therefore recommend that managers and hunting organizations continue their efforts to increase awareness among moose hunters about the increased risk of bear-caused injuries during this time of year, particularly for hunters using unleashed baying dogs. The growing and expanding bear population means that the risk, or chance, of encountering a bear is increasing.

Our results show that half of the bears reduced their activity significantly before they arrived at their den. Bears could therefore be more likely to respond aggressively to disturbance during this time, because of a change in prehibernation behavior rather than because of the presence of a den (resource defense). Therefore, hunters could reduce risk by approaching barking dogs that are assumed to be holding a moose at bay with caution until they are certain that the dog has a moose and not a bear at bay. This has dual benefits in terms of reduced risk of injury to the hunter, as well as to the bear.
