# Supplementary material for: Den Entry Behavior in Scandinavian Brown Bears: Implications for Preventing Human Injuries
Source: J Wildl Manage. 2015 Feb 13;79(2):274–87. doi: 10.1002/jwmg.822 (PMC4383655; doi:10.1002/jwmg.822)
Supplement: Supplementary file 1 [file jwmg0079-0274-sd1.doc]

Supplemental Material

2014-02-27

Sahlén, V. et al. 2014.

Den Entry Behavior in Scandinavian Brown Bears; Implications for Preventing Human Injuries. Journal of Wildlife Management: in review.

Table S1: Variable transformations.

| Variable (definitions available in Main text Table 2) | Transformation Type |
| --- | --- |
| Bear age | log |
| Time spent in den area before hibernation activity | log10 |
| Time spent at den site before hibernation activity | log10 |
| Distance to E45 (European Highway) | log |
| Distance to main road (paved) | log |
| Distance to main dirt roads | square root |
| Distance to medium dirt roads | log10 |
| Distance to railroad | log |
| Distance to town roads | log10 |
| Distance to settlement type 1 & 2 | log10 |
| Distance to settlement type 3 | log10 |
| Distance to settlement type 4 | log 10 |
